# Supplementary material for: Gastro-Resistant Microparticles Produced by Spray-Drying as Controlled Release Systems for Liposoluble Vitamins
Source: Pharmaceutics. 2022 Jul 15;14(7):1480. doi: 10.3390/pharmaceutics14071480 (PMC9319434; doi:10.3390/pharmaceutics14071480)
Supplement: Supplementary file 1 [file pharmaceutics-14-01480-s001.zip › pharmaceutics-1767757-supplementary.pdf]

Gastro-resistant microparticles produced by spray-drying as controlled release systems for liposoluble vitamins

Francesca Terracina[1], Roberto Caruana[2], Francesco Montalbano[2], Mariano Licciardi[1]*

[1] Dipartimento di Scienze e Tecnologie Biologiche Chimiche e Farmaceutiche (STEBICEF), Università degli Studi di Palermo, 90123 Palermo, Italy.

[2] Technology Scientific S.r.l., Viale delle Scienze, Edificio 18, 90128, Palermo, Italy

*Corresponding author: mariano.licciardi@unipa.it; Dipartimento di Scienze e Tecnologie Biologiche Chimiche e Farmaceutiche (STEBICEF), Università degli Studi di Palermo, 90123 Palermo, Italy.

Supplementary Information

Table S1. Summary table of the formulation compositions of all the batches subjected to the spray drying process.

Batches	Formulations′ composition
**A1**	2 g Arabic gum<br>2.5 g Mannitol<br>0.25 g Soy lecithin<br>0.5 g Retinyl palmitate (in 5 ml of ethanol)<br>0.6 g Eudraguard Biotic (2 ml 30% v/w)<br>45 ml Distilled water
**B1**	2 g Arabic gum<br>2.5 g Mannitol<br>0.25 g Soy lecithin<br>0.5 g β-carotene(in 5 ml of ethanol)<br>0.6 g Eudraguard Biotic (2 ml 30% v/w)<br>45 ml Distilled water
**C1**	2 g Arabic gum<br>2.5 g Mannitol<br>0.25 g Soy lecithin<br>0.5 g Retinyl acetate (in 5 ml of ethanol)<br>0.6 g Eudraguard Biotic (2 ml 30% v/w)<br>45 ml Distilled water

**D1**	2 g Arabic gum<br>2.5 g Mannitol<br>0.25 g Soy lecithin<br>0.1 g Cholecalciferol (in 5 ml of ethanol)<br>0.6 g Eudraguard Biotic (2 ml 30% v/w)<br>45 ml Distilled water
**E1**	2 g Arabic gum<br>2.5 g Mannitol<br>0.25 g Soy lecithin<br>0.25 g α-tocopherol (in 5 ml of ethanol)<br>0.6 g Eudraguard Biotic (2 ml 30% v/w)<br>45 ml Distilled water
**A2**	2 g Arabic gum<br>2.5 g Mannitol<br>0.25 g Soy lecithin<br>0.5 g Retinyl palmitate (in 5 ml of ethanol)<br>0.25 g CAP<br>45 ml Distilled water
**B2**	2 g Arabic gum<br>2.5 g Mannitol<br>0.25 g Soy lecithin<br>0.5 g β-carotene (in 5 ml of ethanol)<br>0.25 g CAP<br>45 ml Distilled water
**C2**	2 g Arabic gum<br>2.5 g Mannitol<br>0.25 g Soy lecithin<br>0.5 g Retinyl acetate (in 5 ml of ethanol)<br>0.25 g CAP<br>45 ml Distilled water
**D2**	2 g Arabic gum<br>2.5 g Mannitol<br>0.25 g Soy lecithin<br>0.1 g Cholecalciferol (in 5 ml of ethanol)<br>0.25 g CAP<br>45 ml Distilled water
**E2**	2 g Arabic gum<br>2.5 g Mannitol<br>0.25 g Soy lecithin<br>0.25 g α-tocopherol (in 5 ml of ethanol)<br>0.25 g CAP<br>45 ml Distilled water
**A3**	2.5 g WPs<br>0.5 g Retinyl palmitate<br>50 ml Whole milk
**B3**	2.5 g WPs<br>0.5 g β-carotene<br>50 ml Whole milk

**C3**	2.5 g WPs<br>0.5 g Retinyl acetate<br>50 ml Whole milk
**D3**	2.5 g WPs<br>0.1 g Cholecalciferol<br>50 ml Whole milk
**E3**	2.5 g WPs<br>0.25 g α-tocopherol<br>50 ml Whole milk

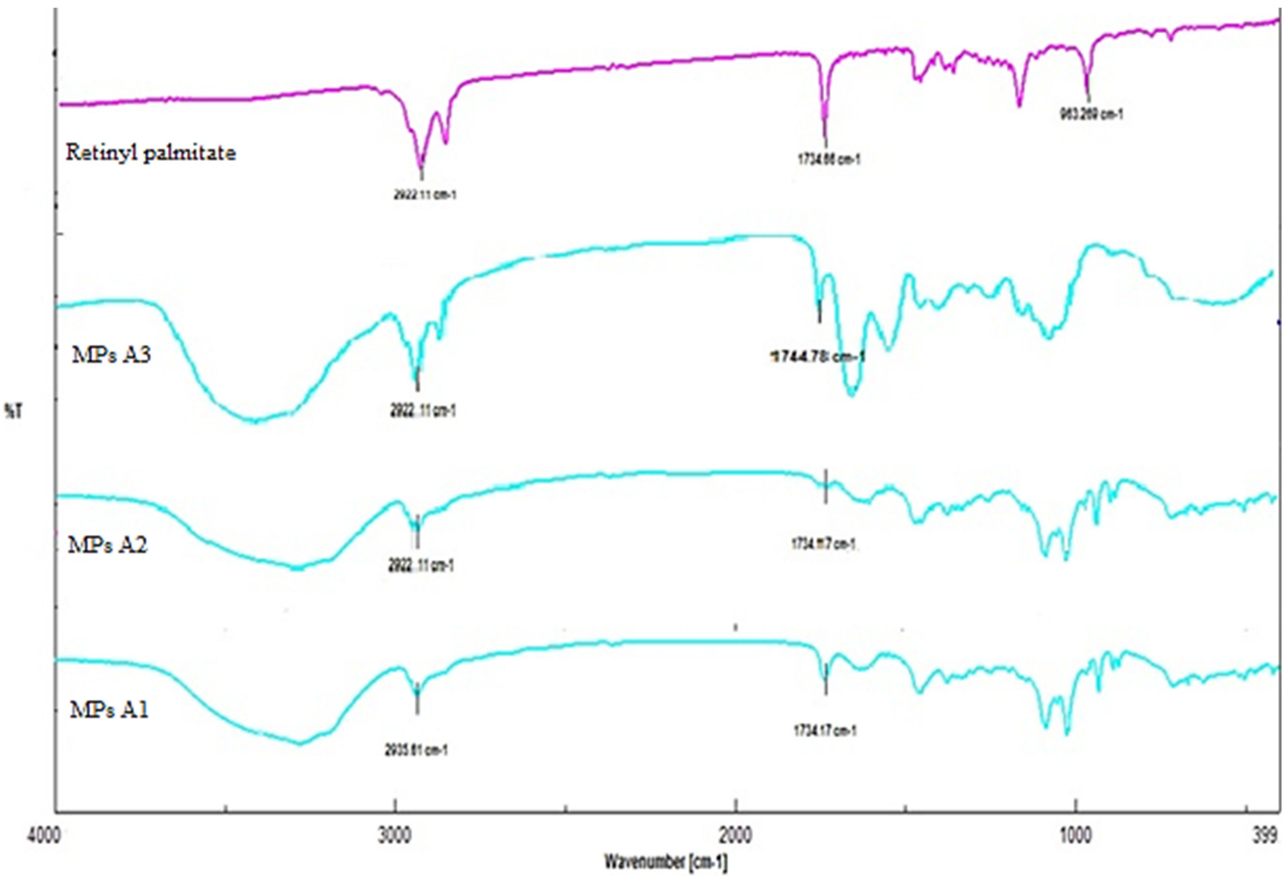

Figure S1. FTIR spectra of pure retinyl palmitate and MP formulations A1 (Eudraguard Biotic), A2 (CAP) and A3 (WPs).

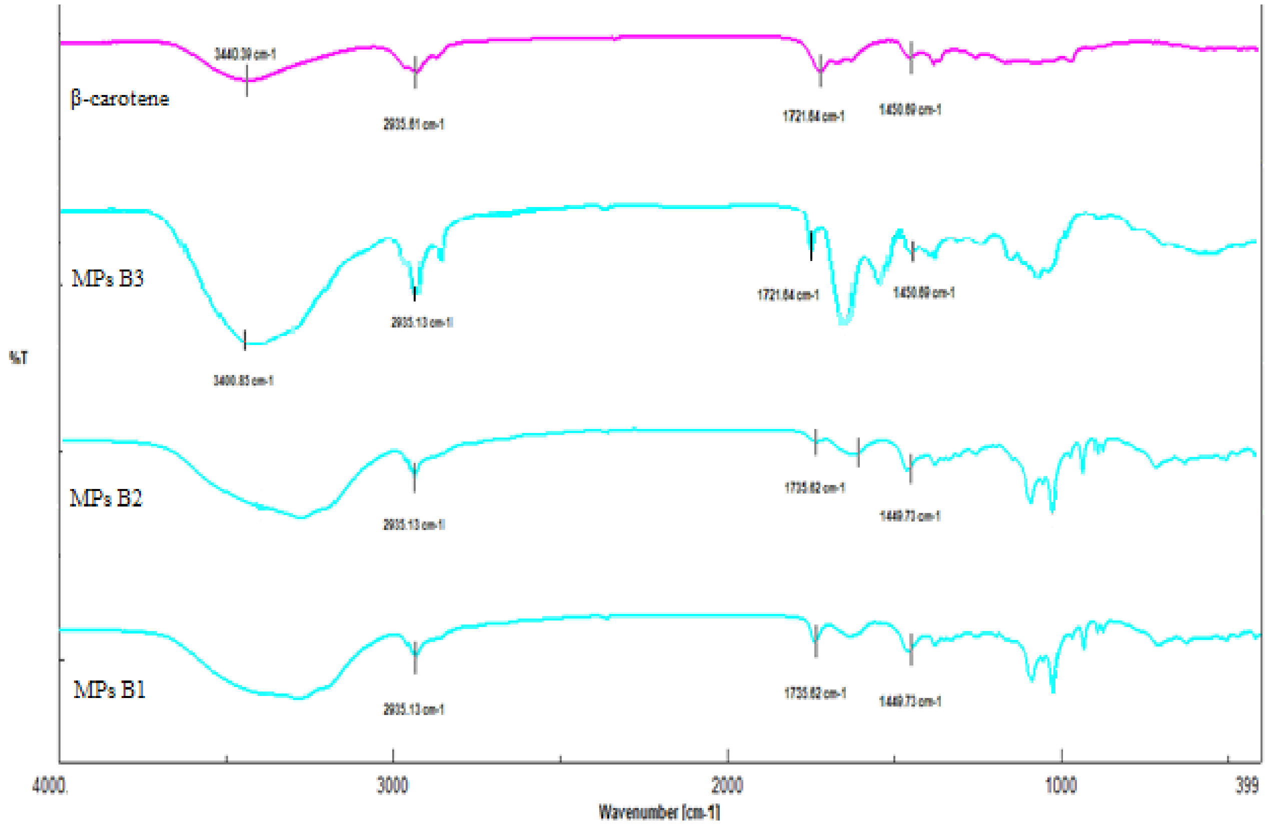

Figure S2. FTIR spectra of pure β-carotene and MP formulations B1 (Eudraguard Biotic), B2 (CAP) and B3 (WPs).

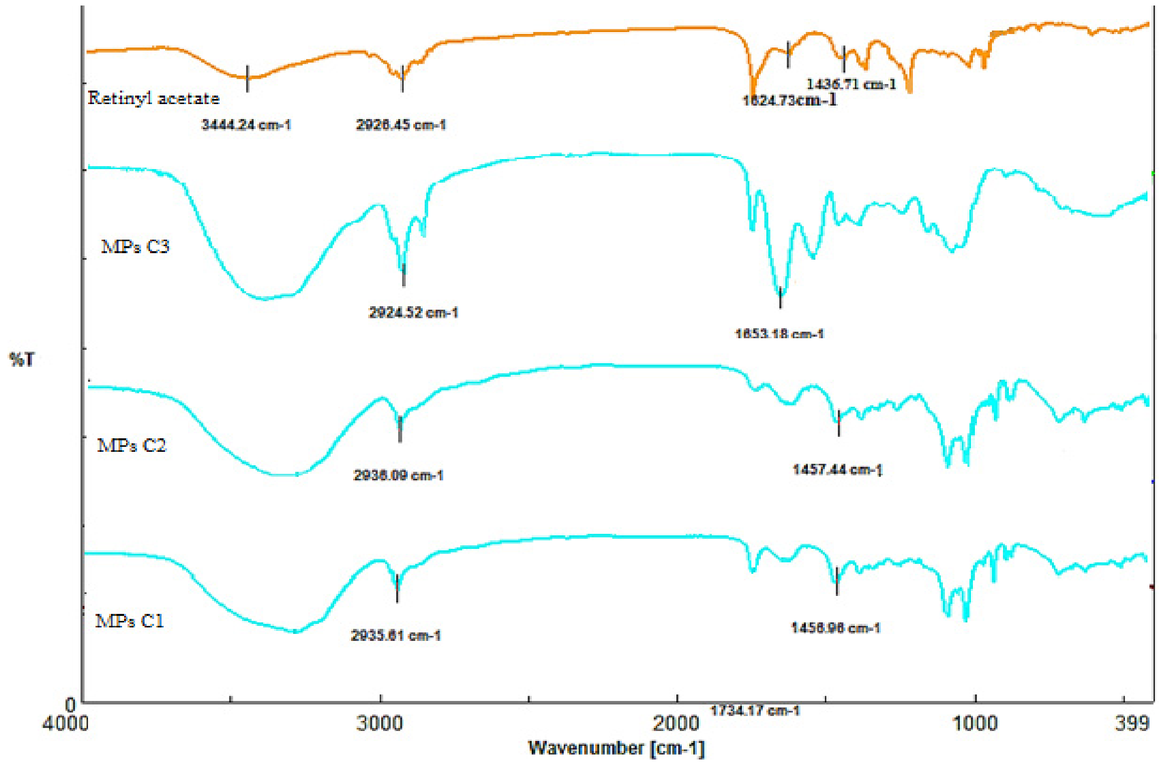

Figure S3. FTIR spectra of pure retinyl acetate and MP formulations C1 (Eudraguard Biotic), C2 (CAP) and C3 (WPs).

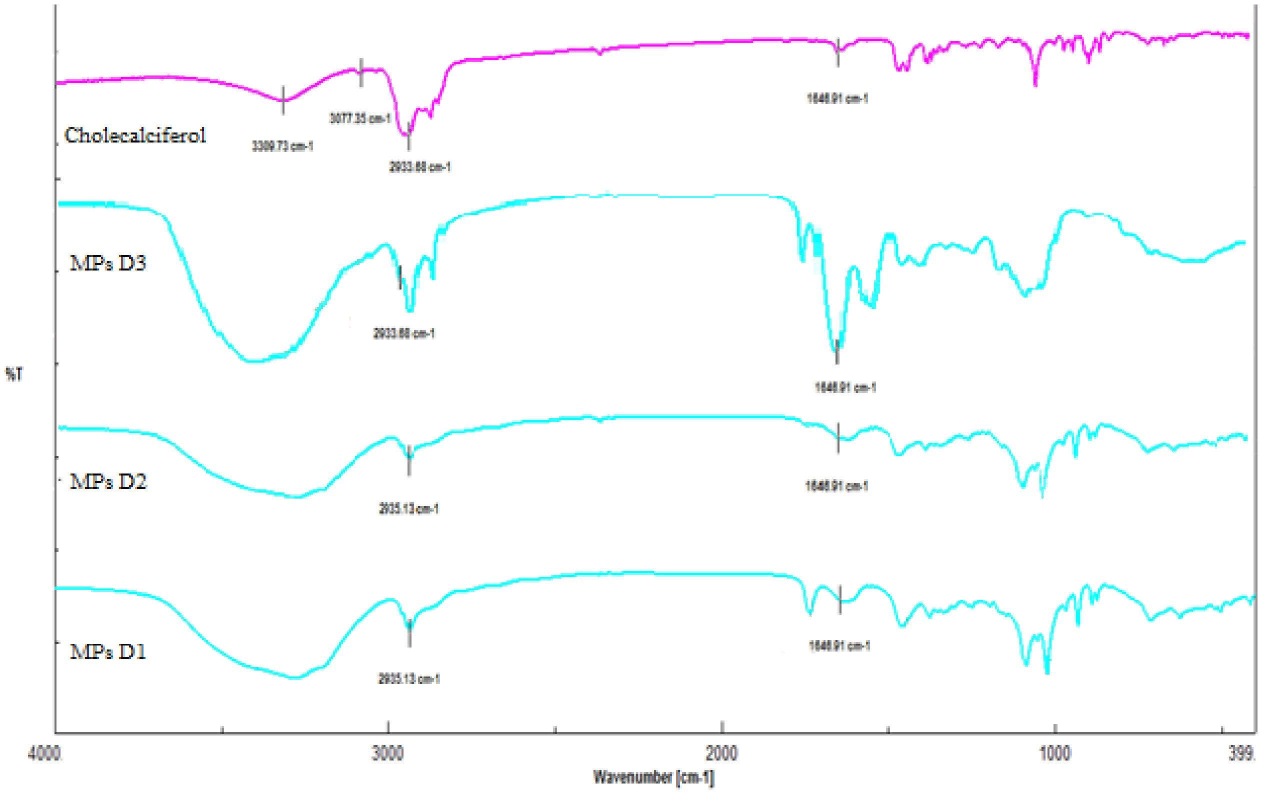

Figure S4. FTIR spectra of pure cholecalciferol and MP formulations D1 (Eudraguard Biotic), D2 (CAP) and D3 (WPs).

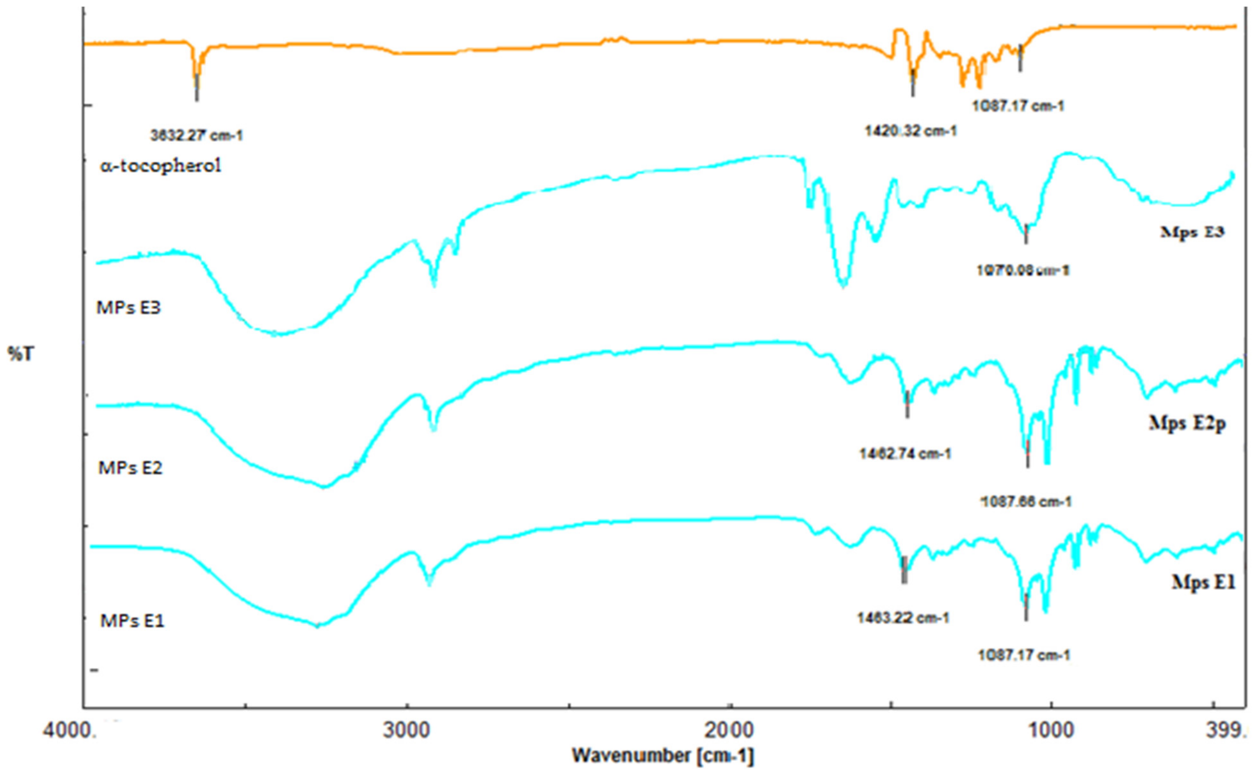

Figure S5. FTIR spectra of pure α-tocopherol and MP formulations E1 (Eudraguard Biotic), E2 (CAP) and E3 (WPs).

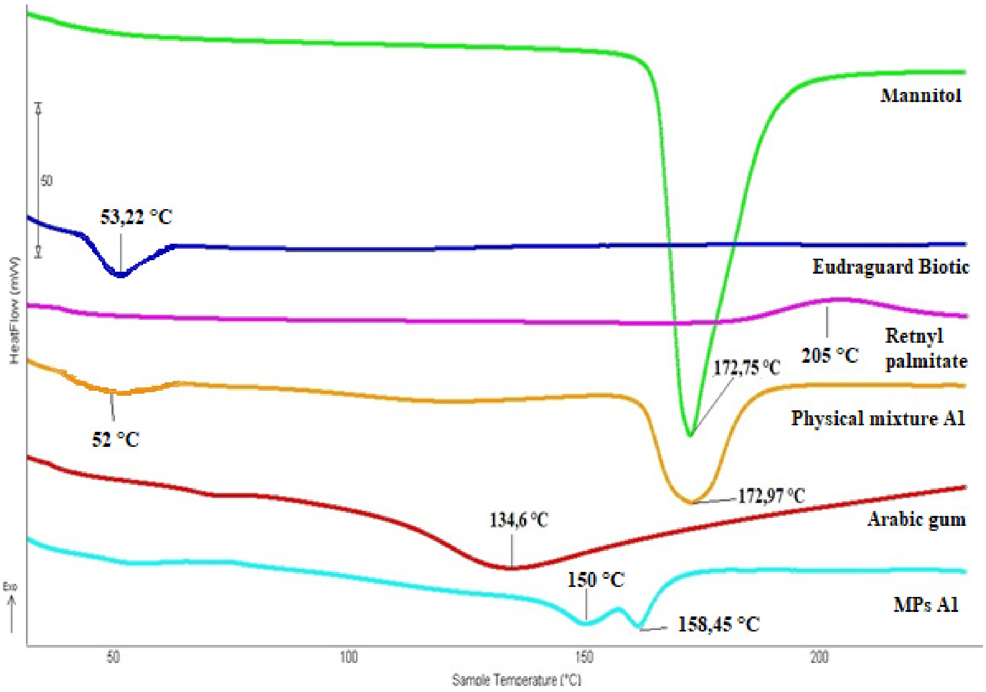

Figure S6. Comparison between the DSC graphs of Mannitol, Eudraguard Biotic, retinyl palmitate, the physical mixture of the compounds presents in the formulation A1 and microparticles (MPs A1).

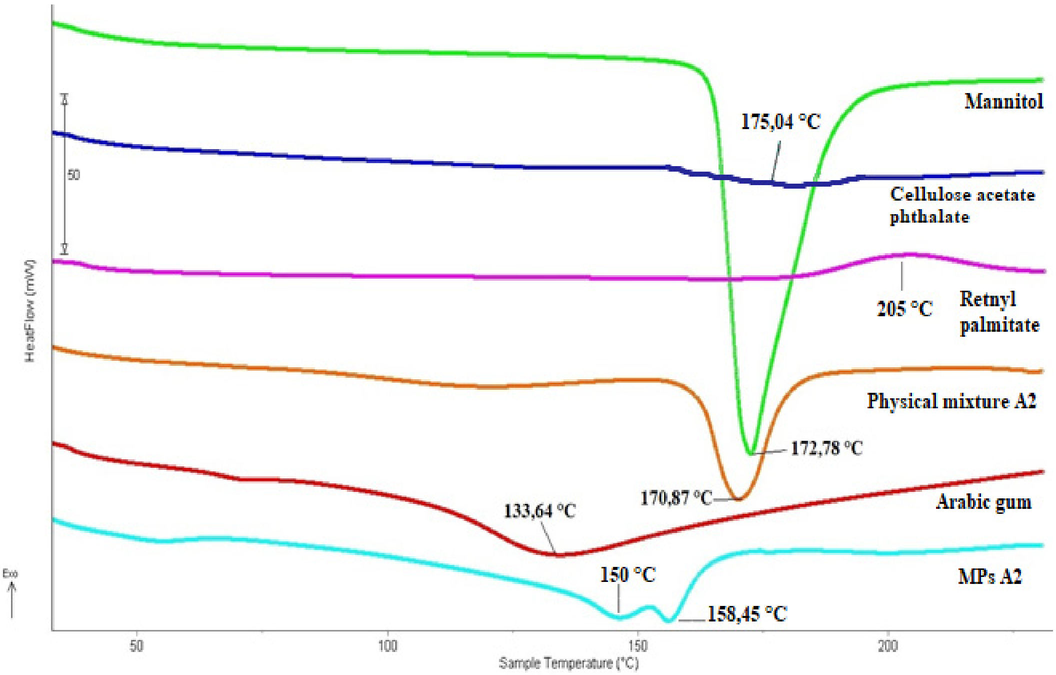

Figure S7. Comparison between the DSC graphs of Mannitol, CAP, retinyl palmitate, the physical mixture of the compounds presents in the formulation A2 and microparticles (MPs A2).

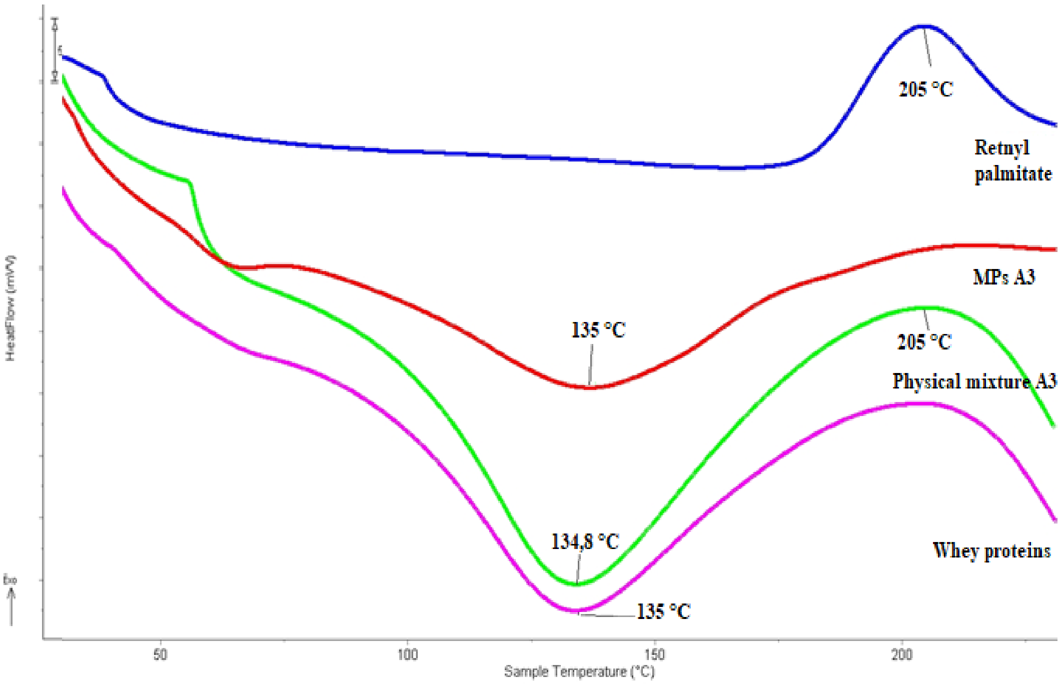

Figure S8. Comparison between the DSC graphs of WPs, retinyl palmitate, the physical mixture of the compounds presents in the formulation A3 and microparticles (MPs A3).

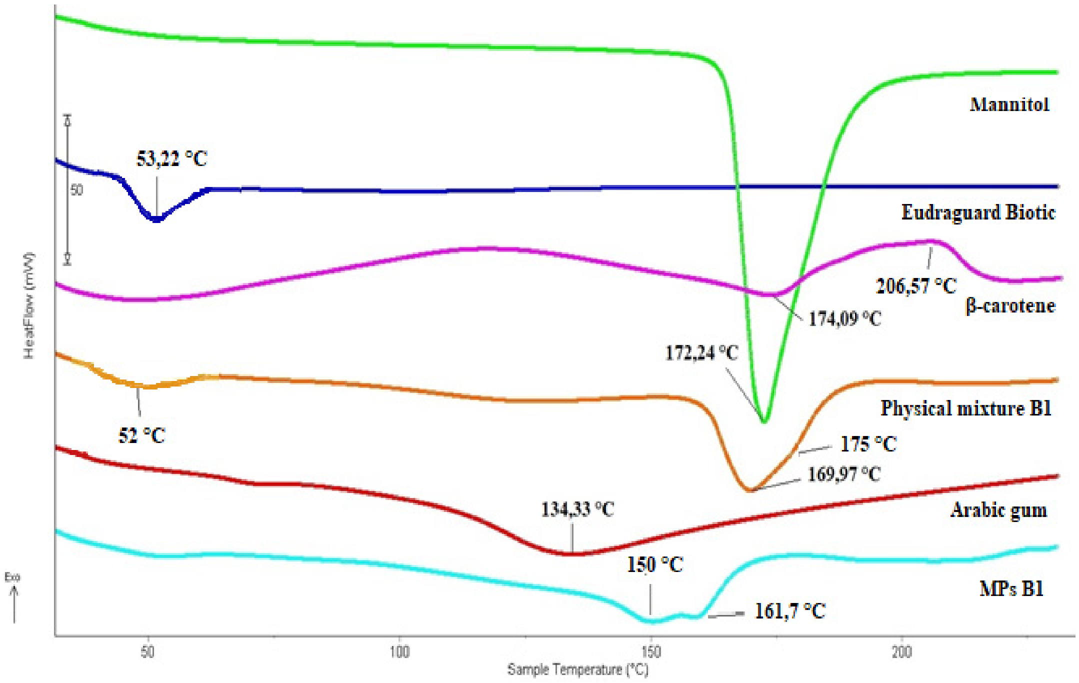

Figure S9. Comparison between the DSC graphs of mannitol, Eudraguard Biotic, β-carotene, the physical mixture of the compounds present in the formulation B1 and microparticles (MPs B1).

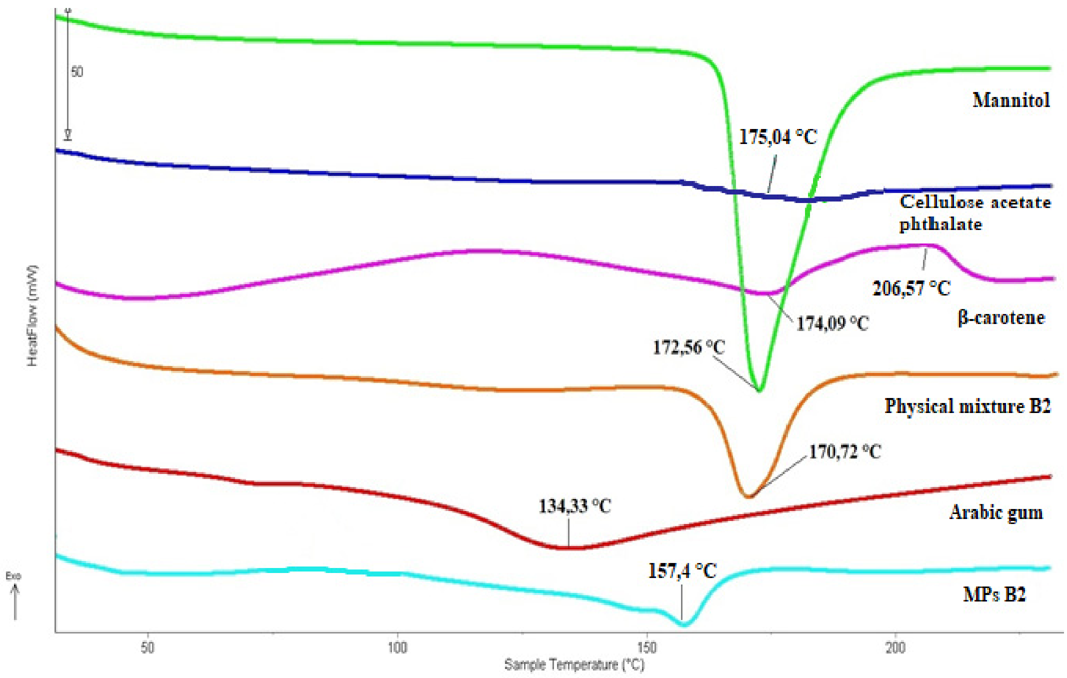

Figure S10. Comparison between the DSC graphs of mannitol, CAP, β-carotene, the physical mixture of the compounds presents in the formulation B2 and microparticles (MPs B2).

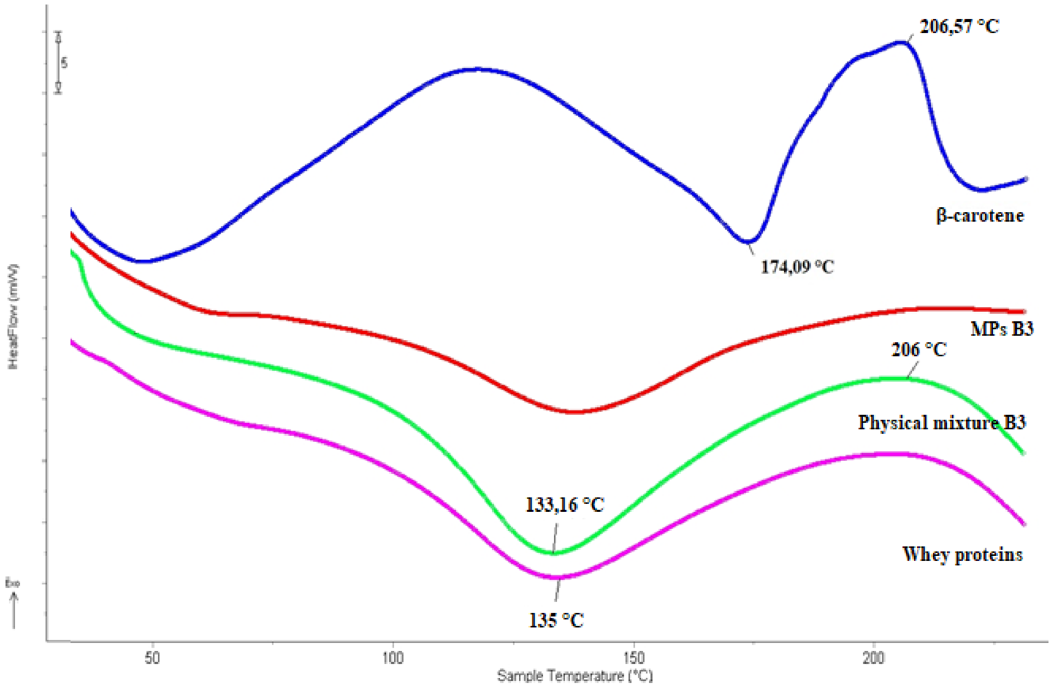

Figure S11. Comparison between the DSC graphs of WPs, β-carotene, the physical mixture of the compounds presents in the formulation B3 and microparticles (MPs B3).

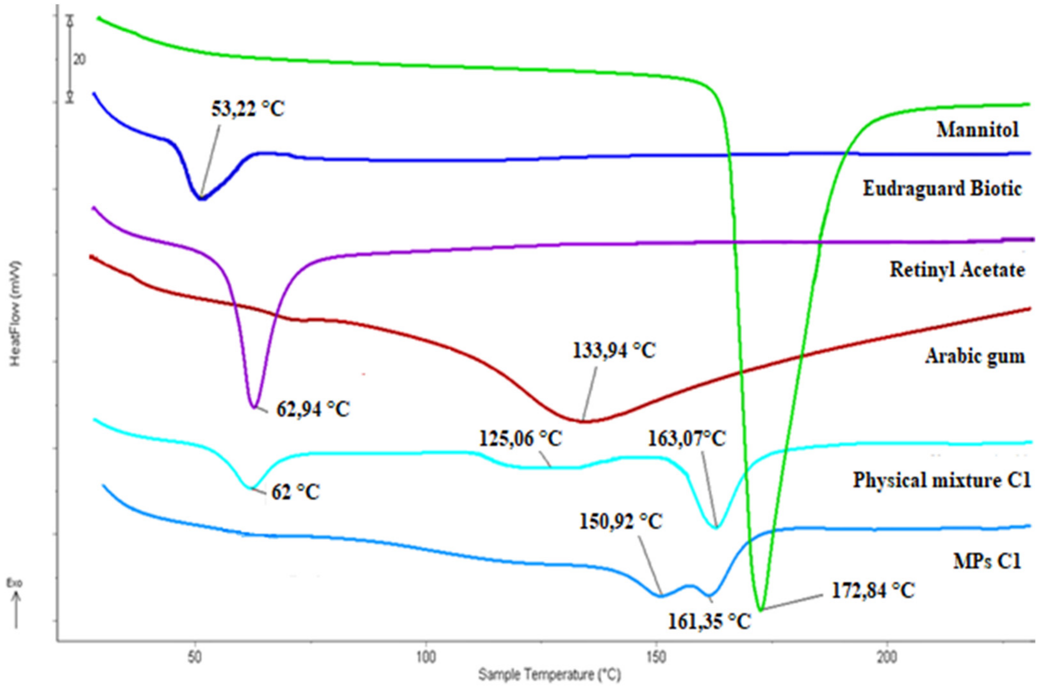

Figure S12. Comparison between the DSC graphs of mannitol, Eudraguard Biotic, retinyl acetate, the physical mixture of the compounds presents in the formulation C1 and microparticles (MPs C1).

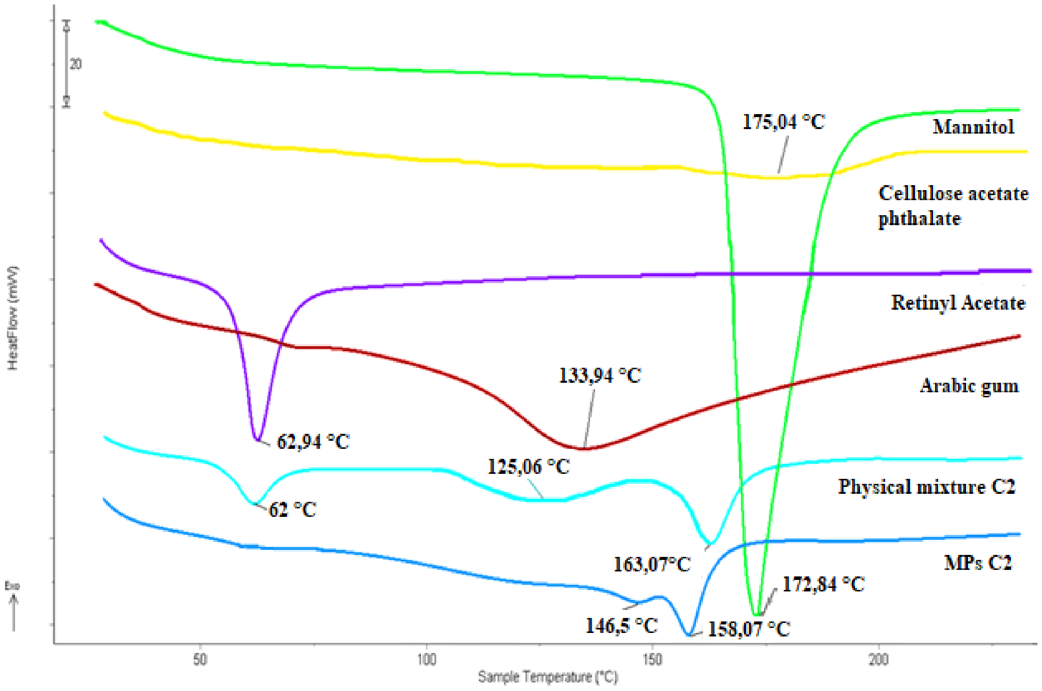

Figure S13. Comparison between the DSC graphs of mannitol, CAP, retinyl acetate, the physical mixture of the compounds presents in the formulation C2 and microparticles (MPs C2).

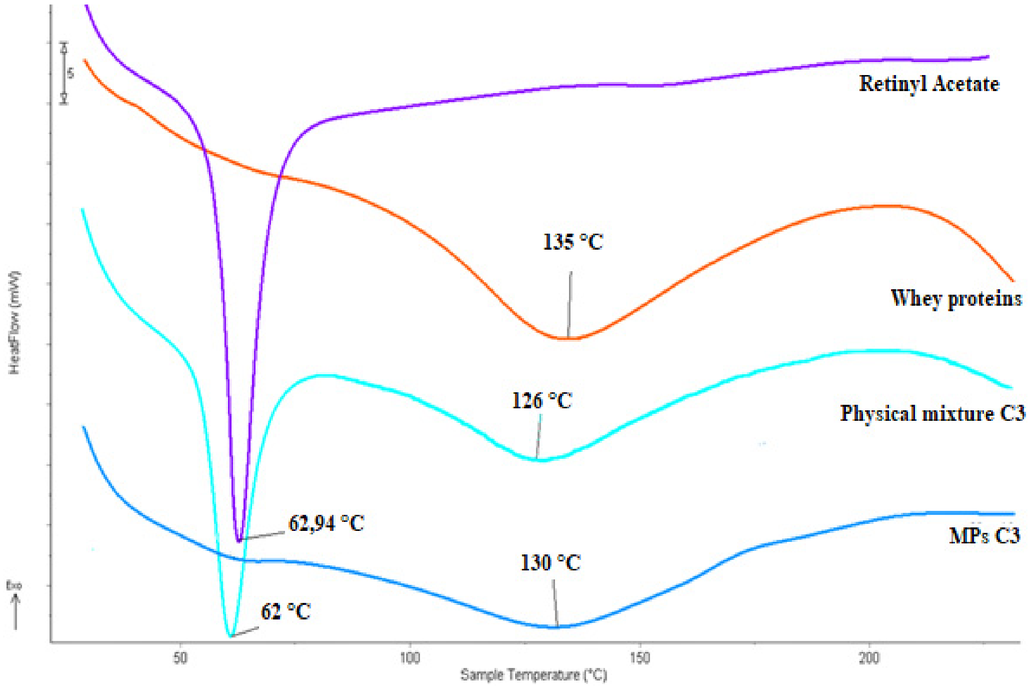

Figure S14. Comparison between the DSC graphs of WPs, retinyl acetate, the physical mixture of the compounds presents in the formulation C3 and microparticles (MPs C3).

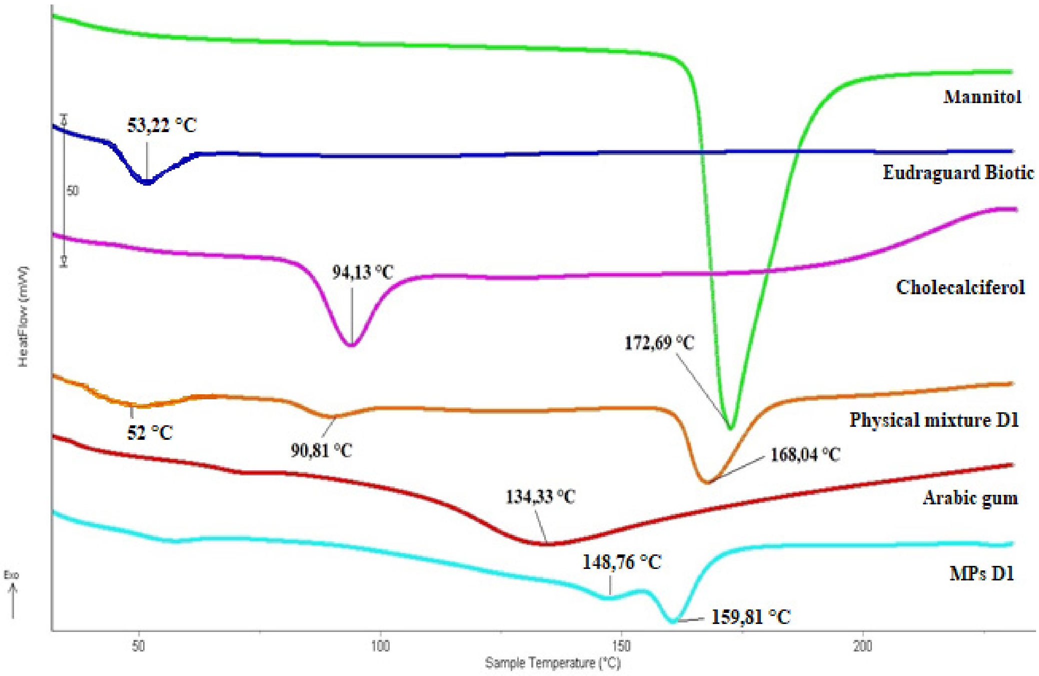

Figure S15. Comparison between the DSC graphs of mannitol, Eudraguard Biotic, cholecalciferol, the physical mixture of the compounds presents in the formulation D1 and microparticles (MPs D1).

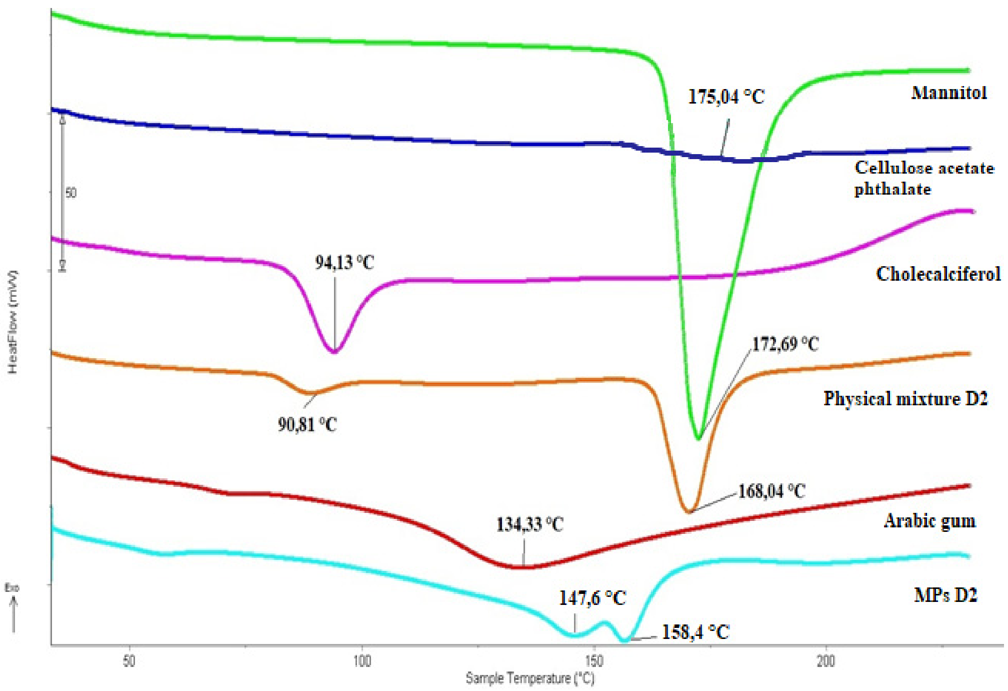

Figure S16. Comparison between the DSC graphs of mannitol, CAP, cholecalciferol, the physical mixture of the compounds presents in the formulation D2 and microparticles (MPs D2).

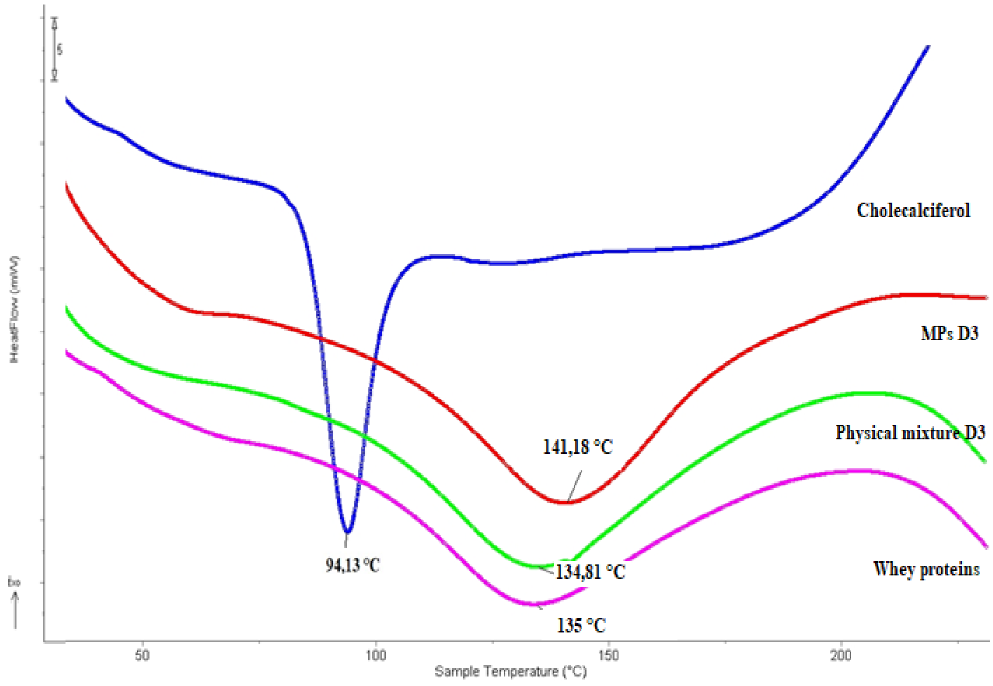

Figure S17. Comparison between the DSC graphs of WPs, cholecalciferol, the physical mixture of the compounds presents in the formulation D3 and microparticles (MPs D3).

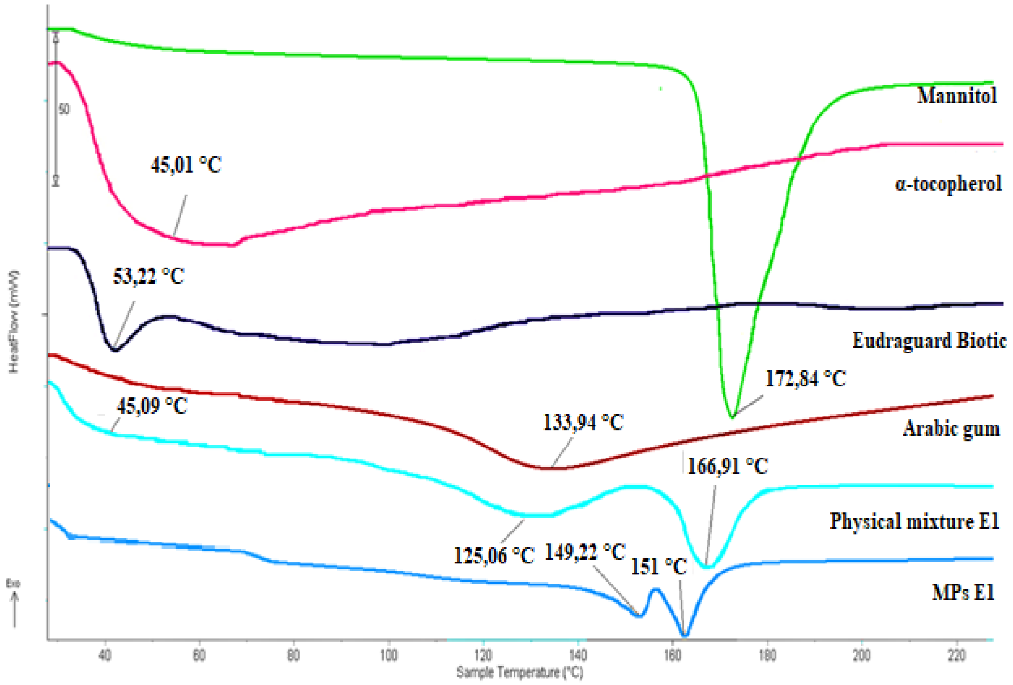

Figure S18. Comparison between the DSC graphs of mannitol, Eudraguard Biotic, α-tocopherol, the physical mixture of the compounds presents in the formulation E1 and microparticles (MPs E1).

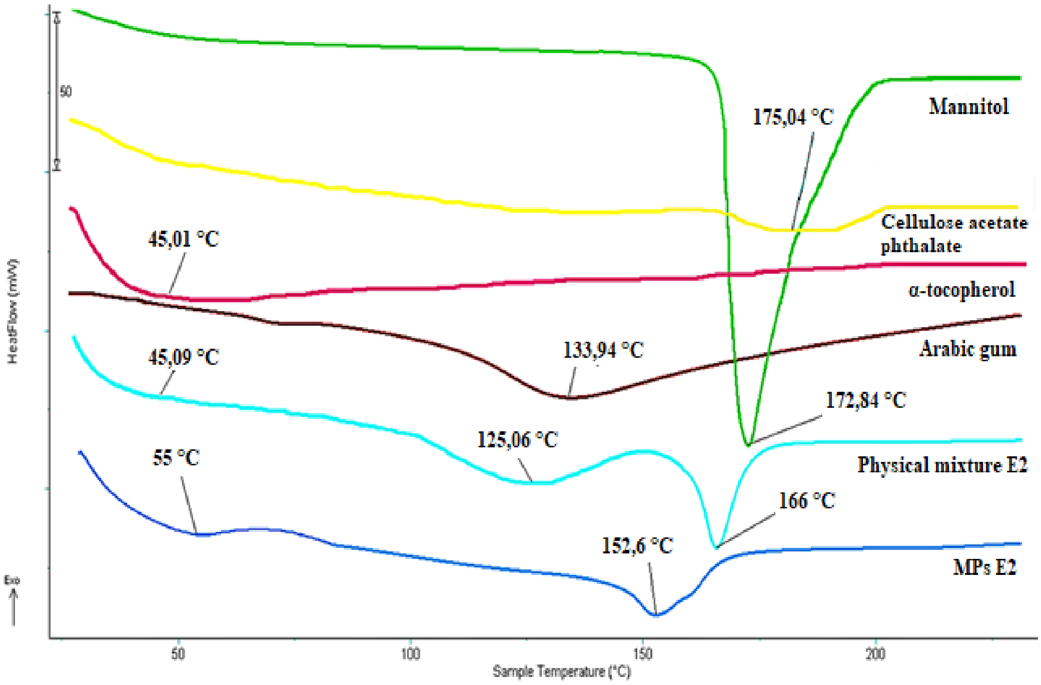

Figure S19. Comparison between the DSC graphs of mannitol, CAP, α-tocopherol, the physical mixture of the compounds presents in the formulation E2 and microparticles (MPs E2).

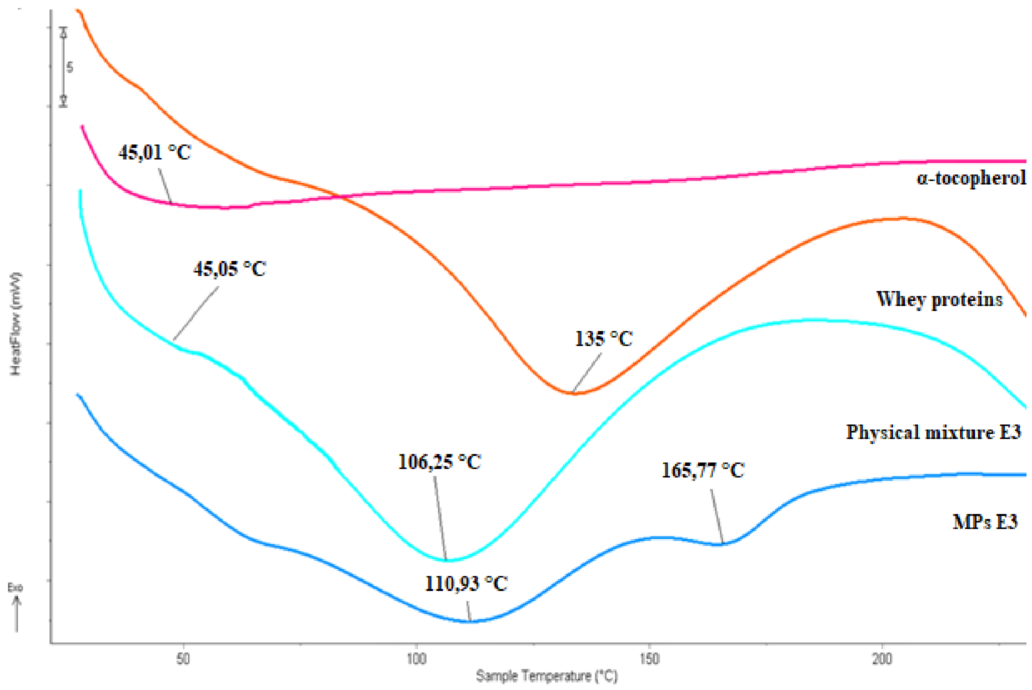

Figure S20. Comparison between the DSC graphs of WPs, α-tocopherol, the physical mixture of the compounds presents in the formulation E3 and microparticles (MPs E3).
